# Health Benefits, Applications, and Analytical Methods of Freshly Produced Allyl Isothiocyanate

**DOI:** 10.3390/foods14040579

**Published:** 2025-02-10

**Authors:** Walaa Alibrahem, Duyen H. H. Nguyen, Nihad Kharrat Helu, Florence Tóth, Péter Tamás Nagy, János Posta, József Prokisch, Csaba Oláh

**Affiliations:** 1 Doctoral School of Health Sciences, University of Debrecen, Egyetem tér 1, 4028 Debrecen, Hungary; nihad.kharrat.helu@mailbox.unideb.hu; 2 Faculty of Agricultural and Food Sciences and Environmental Management, Institute of Animal Science, Biotechnology and Nature Conservation, University of Debrecen, Böszörményi Street 138, 4032 Debrecen, Hungary; nguyen.huu.huong.duyen@agr.unideb.hu (D.H.H.N.); jprokisch@agr.unideb.hu (J.P.); 3 Faculty of Agricultural and Food Sciences and Environmental Management, Institute of Water and Environmental Management, University of Debrecen, Böszörményi Street 138, 4032 Debrecen, Hungary; toth.florence@agr.unideb.hu (F.T.); nagypt@agr.unideb.hu (P.T.N.); 4 Health Care Service Units, Diagnostic Units, Forensic Medicine, University of Debrecen Clinical Center, University of Debrecen, Nagyerdei körút 98, 4032 Debrecen, Hungary; posta@med.unideb.hu; 5 Mathias Institute, University of Tokaj, Eötvös Str. 7, 3950 Sárospatak, Hungary; olahcs@gmail.com; 6 Neurosurgery Department, Borsod County University Teaching Hospital, Szentpéteri kapu 72-76, 3526 Miskolc, Hungary

**Keywords:** allyl isothiocyanate, horseradish, antioxidant, anti-inflammatory, antibacterial, anticancer capabilities, apoptosis, cardiovascular health, food preservation

## Abstract

Allyl isothiocyanate (AITC) is a low-molecular-weight natural chemical predominantly obtained from the autolysis of sinigrin, a glucosinolate found in cruciferous vegetables like mustard, horseradish, and wasabi. AITC has sparked widespread interest due to its various biological actions, which include strong antioxidant, anti-inflammatory, antibacterial, and anticancer capabilities. This compound offers promising potential in several fields, particularly in food preservation, medicine, and enhancing food quality through natural means. AITC’s effectiveness against a broad spectrum of microorganisms, including foodborne pathogens and spoilage agents, makes it an attractive natural alternative to synthetic preservatives. The potential to extend the shelf life of perishable foods makes AITC an important tool for food production, meeting rising customer demand for natural additives. In addition to its antimicrobial effects, AITC demonstrates significant anti-inflammatory activity, reducing levels of pro-inflammatory cytokines and modulating key signaling pathways, which could make it valuable in managing chronic inflammatory conditions. Furthermore, emerging research highlights its potential in cancer prevention and treatment, as AITC has been demonstrated to induce apoptosis and inhibit cell increase in several cancer cell lines, offering a natural approach to chemoprevention. This review delves into the chemical structure, metabolism, and bioavailability of freshly produced AITC, providing a comprehensive overview of its beneficial properties. Challenges related to AITC’s volatility, dosage optimization, and regulatory considerations are also discussed, alongside future research directions to enhance the stability and efficacy of AITC-based formulations. The findings underscore AITC’s role as a versatile bioactive compound with known potential to support human health and the sustainable food industry.

## 1. Introduction

Allyl isothiocyanate (AITC) is a natural low-molecular-weight chemical that is a well-known byproduct of sinigrin autolysis [1]. AITC exhibits a wide spectrum of health properties, such as antioxidant, anti-inflammatory, antibacterial, antifungal, antiangiogenic, neuroprotective, or analgesic benefits [1]. The active compound can also enhance the function of detoxification enzymes in the body, as well as a strong influence on the bioaccessibility of nutrients in the human gastrointestinal system [2]. Its role in improving cardiovascular health has also been noted, with studies suggesting that AITC may improve endothelial function and lower the risk of cardiovascular diseases [1]. Based on previous research, AITC may be beneficial in human nutrition, and its mild spiciness ensures that it replaces synthetic additives in food such as Monosodium Glutamate (MSG) [3]. AITC found in mustard, horseradish, and wasabi, boasts a rich history extremely connected with human civilization [4]. These plants have existed as cookery staples for centuries, adding taste to dishes internationally [4]. Further than their culinary roles, they have also held an important place in traditional medicine as stimulants to improve circulation and absorption, as antiseptics to treat wounds and infections, as pain relievers, and as remedies for respiratory ailments. Researchers are becoming more interested in natural compounds, especially their wide range of effects on human health [5]. Researchers have further explored the several uses of AITC in food manufacturing, including its use as a flavoring agent and its potential synergistic effects when combined with other natural compounds [3]. AITC is an oily, bright-yellow liquid that gives horseradish its head-clearing taste and aroma during the roasting process [5]. Human experience of AITC is certainly common and frequent, as many common cruciferous vegetables are a rich source of AITC, but the experience levels have not been well-documented [6]. AITC has been used historically as a means to improve meat quality, constraining the deleterious effects of certain bacteria [6]. Food contamination with these Gram-negative bacteria has been the subject of interest for over a century [7]. Recently, the effectiveness of AITC in inhibiting the activity of mycotoxins in stored barley was demonstrated [2]. This essay’s objectives are to review several health properties and applications of freshly prepared AITC and to explore the properties of this compound [6]. Research on the properties of this compound is of great scientific interest because it may result in the possible use of AITC in medicine or the food-processing industry [8].

**Figure 1:**
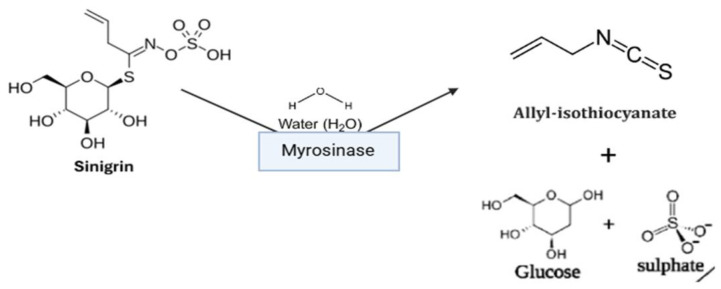
Sinigrin is converted enzymatically into AITC, glucose, and sulfate.

AITC is found in cruciferous vegetables and it has a relatively simple molecular design consisting of carbon, hydrogen, nitrogen, and sulfur, along with the odd and unique linkage of the allyl radical inserted between sulfur and nitrogen of the SCN moiety (Figure 1) [9]. The presence of the allyl is the major reason associated with AITC for its unique and phenomenal characteristics [2]. This simple molecule can function as both a base and a nucleophile during chemical reactions. This natural gem, released from the plant source, is notably reactive and metabolized by microorganisms and eukaryotes once consumed, expelled as volatiles from active biomass, and interacts with various food systems and biological elements [10]. The unique properties of AITC make it an inspiration for such vital applications, including flavor formation through Maillard browning, biopreservation, disinfection compounds, and pharmaceuticals [11]. Inherent volatility, its aroma action, as well as other sensory properties, provide additional benefits to the above qualities of AITC [12]. At very high concentrations it also sometimes overrides the flavor and aroma aspects, making it an effective tool in pest control [5]. For these diverse applications, understanding the basic chemical structure and reactive properties of AITC is exciting and crucial [13]. Being the key aspect of the biological activities, including species-specific activity, nutritional, and pharmacological relevance of this natural compound, allyl isothiocyanate has been at the center of research endeavors [14].

## 2. Biological Activities and Health Benefits of Allyl Isothiocyanate

AITC is an essential component of mustard, horseradish, radish, and wasabi, It is the substance that gives these foods their features of a sharp taste and smell [3,15]. Freeze-drying preserves the antioxidant properties better, while hot-air drying at higher temperatures enhances antioxidant activity [16]. In this section, we highlight the most studied activities of this compound, together with some of its pharmacological applications [16]. The spectrum of pathogenic and spoilage agents that AITC has been proven to be effective against ensures its broad-spectrum potent antimicrobial activity [17,18]. Oral administration of AITC inhibited the growth of total aerobic bacteria, including coliforms and Clostridia, in rats [19,20]. AITC showed excellent inhibitory efficacy against food pathogens in the rat and poultry gut microflora [21]. Furthermore, it showed high antimicrobial activity against fruit-spoilage bacteria, directionally restraining rapid spoilage and extending the shelf life of fruit [22,23] (Table 1).

Another important effect of AITC is its anti-inflammatory activity [24]. In this context, AITC reduced inflammation in rodents. Especially, AITC suppresses the internalization of vascular endothelial cadherin in vitro, and can also prevent the expression of TNF-α, IFN-γ, IL-4, and IL-17 in splenocytes [25,26]. AITC also suppressed serine phosphorylation of IκB [27]. It is widely acknowledged that AITC exhibits anticancer activity [28]. A study demonstrated that AITC could inhibit cancer stem cell-like characteristics in cells by dominating the self-renewal and expression of cancer-initiating cell surface markers alongside signaling regulators. This compound may have an impact on colon stromal cells and submucosa [29]. AITC contributes to chemoprevention by preventing the growth of colon polyps as a pan-PPAR agonist in mice [30]. AITC has a direct effect on the upper gastrointestinal tract, and on the mucosa, by inhibiting the progress of swelling [31]. Extensive studies have shown the cardiovascular benefits of AITC in various models, with the protection of endothelial function in rats where it was used prophylactically [32,33].

### 2.1. Antimicrobial Properties

One of the newly developing applications of AITC is its potential as a natural preservative [19]. There is a growing number of studies that demonstrate the effectiveness of AITC-like compounds on a broad range of microbes, including bacteria and fungi [34]. An extremely important point when assessing these studies is the wide range of inhibition employed against microorganisms, from foodborne Gram-negative or Gram-positive bacteria to a wide range of fungi and yeasts [35,36]. It has been reported that AITC, covering the gaseous phase of essential oil, can impair the growth of Listeria monocytogenes, Campylobacter spp., Bacillus cereus, Escherichia coli, Salmonella spp., and Staphylococcus aureus (Table 2) [37,38]. Likewise, there is a broad range of inhibition of fungal activity, as supported by several studies documenting AITC’s efficiency in preventing mold growth on food and food-processing surfaces [39,40]. The basis of inhibition is a disruption of cellular-membrane potential and an alteration of at least seven vital enzymes detectable mostly in 48 h of growth, two of great importance for its inhibitor role made in vitro: dehydrogenase reductase-1 and aminopeptidase [41,42].

AITC can be applied in many matrices to inhibit pathogenic microorganisms in food. The possible commercial applications of AITC in suppressing the growth of undesirable microorganisms in food facilitate its storage [34,35]. Other unstable compounds, such as citral, linalool, and hydrocinnamic acid, have a harmonized limit of 0.002 mg/kg established for food products, which already supports the existence of the compound in food [37,43]. Further investigations support the possible synergistic effect with other antimicrobial compounds [44]. Moreover, several regulatory limitations should be respected, and studies should be conducted to establish a standard in products other than pure AITC [45,46].

### 2.2. Anti-Inflammatory Effects

Inflammation is the body’s natural protective reaction to injury or infection, but when it persists, it can influence the evolution of hypertension, heart diseases, atherosclerosis, stroke, arthritis, and cancer-cell-growth development [47,48]. It is believed that because of these effects, AITC could be very relevant to ongoing efforts in health promotion [3,6]. The potential for including AITC in the diet, either as a cruciferous vegetable or as a health supplement, is also relevant [51]. It has notable effects and is particularly useful in diseases in which inflammation is important, such as autoimmune arthritis [50,51].

**Figure 2:**
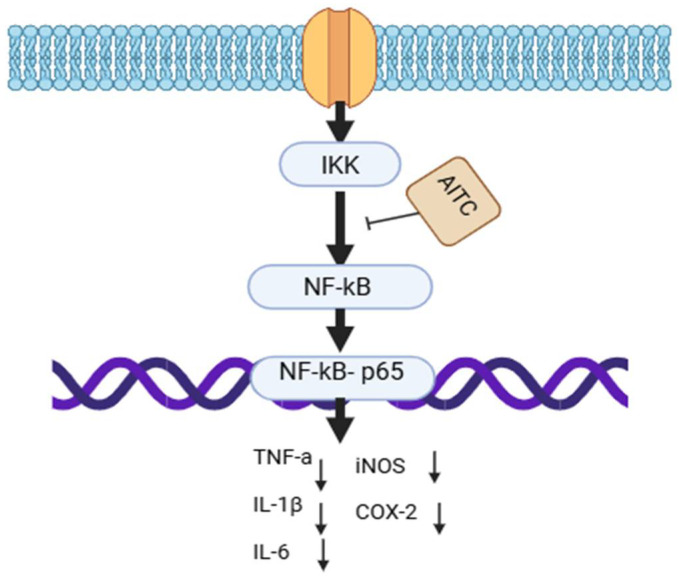
When cells are activated by pro-inflammatory stimuli, IκB kinase (IKK) undergoes activation. This activation allows NF-κB to enter the nucleus, where it starts the transcript of different pro-inflammatory genes (TNF-a, iNOS, Cox-2, IL-6, and IL-1β). NF-kB-P65 activation is inhibited by AITC which leads to a decreasing number of various pro-inflammatory cytokines. AITC: Allyl isothiocyanate. NF-κB: Nuclear factor kappa-light-chain-enhancer of activated B cells. TNFα: Tumor necrosis factor-alpha. IKK: IκB kinase. IL-6: Interleukin-6. IL-1β: Interleukin-1 beta. COX-2: Cyclooxygenase-2.

AITC has been shown to decrease the levels of the the pro-inflammatory cytokine TNF-alpha in lipopolysaccharide (LPS)-induced inflammation in rats [52]. Studies have detected the binding of AITC to cysteine or non-catalytic cysteine regions of proteins and its interaction with signaling molecules, including NF-kappa B, COX-2, and inducible nitric oxide synthase (INOS) [53] (Figure 2). AITC may have immunosuppressive effects on both humoral and cell-mediated immune responses; a single study demonstrated a reduction in dendritic cells in the gut but an increase in the number of macrophages [54]. Stimulation of the immune response has also been reported [47]. Most studies have supported a reduction in pro-inflammatory mediators [53]. Other drugs with anti-inflammatory activity generally act on similar targets, but they involve steroidal anti-inflammatories and non-steroidal anti-inflammatory agents [52]. Given this, logically, AITC also might have the potential for use in inflammation [48].

### 2.3. Anticancer Potential

**Figure 3:**
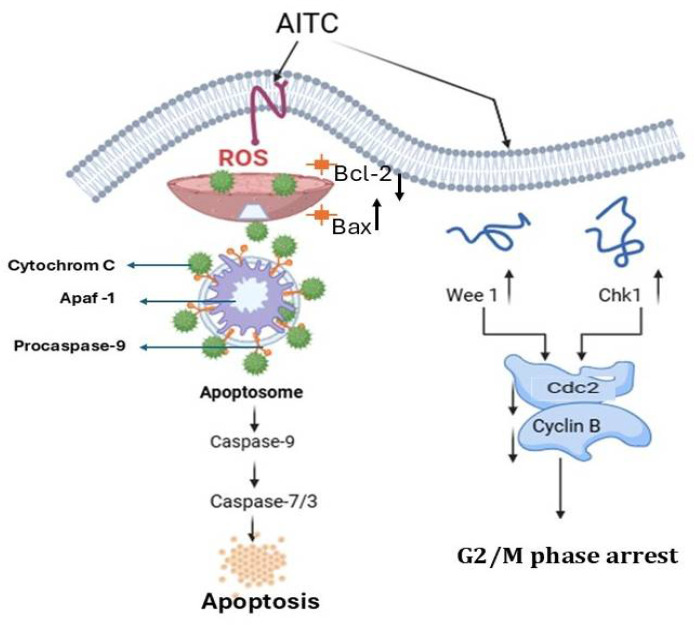
Schematic diagram of the molecular mechanisms for anticancer activity of AITC. AITC is known to exert anticancer effects by stimulating apoptosis or the interplay among G2/M phase arrest. ROS production induced by AITC fully facilitates mitochondrial apoptotic mechanisms. Blockage of G2/M phase by AITC occurs by modulating Wee1, Chk1, and CDK1/cyclin B signal molecules. AITC, allyl isothiocyanate; ROS, reactive oxygen species; *Bcl-2*, B cell lymphoma 2; *Bax*, Bcl2-associated X protein; Cdc2, cyclin-dependent kinase 1; Chk1, checkpoint kinase 1; Apaf-1, apoptotic protease activating factor1; Wee1, a protein kinase.

There are many studies suggesting the anticancer potential of AITC. One of the main methods believed to be responsible for the positive effects of AITC against specific cancer cells is apoptosis induction [55,56]. Indeed, AITC has been proven to effectively induce programmed cell death in many different cancer cell lines from gastrointestinal and hepatic origin [57,58]. In addition to apoptosis induction, available evidence suggests that AITC can modulate the cell cycle of cancer cells, induce cell cycle arrest in the G2/M phase, and suppress the proteins responsible for the G2/M transition, Cdc2 and Cyclin B1 [48,59]. Expression of AITC-triggered apoptosis-related molecules has also been reported, notably downregulation of Bcl-2 and upregulation of Bax [59,60] (Figure 3).

One of the most studied types of cancer with AITC is lung cancer. Since AITC is volatile and irritative for the respiratory system, the question of whether AITC exposure may have tumorigenic outcomes was raised [61]. In vitro studies have given some promising results, indicating that AITC might interfere with the carcinogenesis process, but further studies are warranted [62]. It is unclear from population studies whether allyl compounds or cruciferous vegetables, in general, have protective effects against lung cancer or, rather, whether a high intake of allyl compounds might lead to lung damage, inflammation, and carcinogenesis. AITC has also been studied in the context of colorectal cancer [47,59]. Available studies are limited and suggest that AITC has potential as a preventive agent of colorectal cancer, but at concentrations that are higher than what can be realistically obtained from dietary sources [48]. Whether AITC might prove therapeutically useful in colorectal cancer remains to be investigated. Perhaps for the same reasons of dosage and bioavailability, the characteristics of most available studies are, at best, suggestive of what might be the effects of dietary exposure to AITC [9]. Populations exposed to higher levels of inhaled AITC for professional reasons, rather than dietary, might be more at risk of unwanted toxic effects than protective ones [57]. Larger and more comprehensive studies on cancer patients are warranted to further investigate the possible use of AITC in an integrative treatment of cancer [56,63].

### 2.4. Cardiovascular Benefits

Consumption of AITC may be linked to cardiovascular benefits. Preliminary studies have demonstrated an increase in endothelial function following AITC consumption of 0.5–2.4 mg in various populations, including older healthy adults and smokers. Endothelial function is often impaired in individuals with low cardiovascular health and can be enhanced by lifestyle factors, such as 30 min of aerobic exercise. In this context, AITC may exert a similar effect to food-based supplements to combat endothelial dysfunction [2,7].

AITC is cardioprotective, as demonstrated in animal studies, which observed a reduction in blood pressure and an ability to halt atherosclerotic lesion progression, as well as reduce cholesterol accumulation in rabbit and rat models, and lower serum cholesterol, LDL cholesterol, and triglyceride levels in mice [1,8]. Some of the mechanisms contributing to its cardiovascular benefits may include its antioxidant effects, anti-inflammatory properties, and inhibition of platelet aggregation [64,65].

Additional research has shown various positive effects, including a reduction in hypertension, improved fasting insulin, improved lipid profiles, reduced subcutaneous and visceral white adipose tissue, decreased insulin resistance, and reduced HbA1c and blood glucose levels in both diabetic and healthy animals [28,66].

There is a potential role of AITC in public health to lower the chance of cardiovascular illnesses in high-risk populations [67]. The consumption of AITC through a variety of foods and mustards is also supported by the ubiquity of Brassicaceae in the human diet worldwide [68]. However, before providing any recommendations to the public, studies are needed to determine the probable toxicological implications of AITC consumption [69].

Furthermore, research is needed to decide the optimal dosage or quantity of AITC for preventing age-related chronic diseases such as cardiovascular disease [70,71]. Ingesting a known dosage of AITC from fresh vegetables and/or condiments may also maximize its effects, particularly for common Canadian and global households [72]. Expanding this area of study may lead to research exploring therapeutic applications of AITC in a clinical setting [8,72].

## 3. Sources and Production Methods of Allyl Isothiocyanate

Natural sources of AITC have been traditionally used for centuries and remain relevant today [76]. Even though AITC can be synthetically produced, it is still predominantly supplied directly from natural sources using extraction or distillation, or made in the natural source once requested using enzymatic synthesis [77]. AITC occurs naturally in cruciferous vegetables belonging to the genus Brassica and is mostly in charge of giving mustard and horseradish their flavor [78]. These vegetables are roasted and processed as food additives or dietary supplements in the form of powdered seeds, leaves, or roots [79]. They have long been noted for their beneficial effects on human health as herbal medicine or diet modifications [80]. Natural AITC is available in industrial quantities, and a couple of preparation methods are available, such as solvent extraction and steam distillation of cruciferous seeds, cold pressing of their seeds and leaves, and enzymatic synthesis using the seeds [11,81].

One of the oldest ways of isolating AITC is by the distillation of a relatively large amount of hydrated mustard seed or rapeseed with large quantities of water [82,83]. This approach provides an AITC yield of 7–10% per 100 g of seed [13]. In addition to essential oils, seeds of several species from Sinapis, Brassica, and Sinigrin genera contain relatively large amounts of AITC, sinigrin, enzymatically generated AITC, and allyl nitrile [36,84]. The seed oil-producing and seedcake industries have been developed for these species, and enormous amounts of these materials have become available [16]. Therefore, these species are currently the main natural sources of AITC [85]. A less paternal process to obtain AITC is by dry distillation of the seeds above 600 °C [86]. AITC is also a natural product contained in the seed and root of horseradish, which contains up to 98% 2-propenyl and 30–33% diallyl isothiocyanate [87]. That product can be sold to retail in powder, stick, or liquid form [82].

### 3.1. Natural Sources

AITC is abundant in many cruciferous vegetables [47]. These include different types of edible Brassicas, such as white mustard, Chinese mustard, brown mustard, and turnip [11,12]. They have been considered reliable natural sources of AITC within many historical cuisines, such as in Western Europe, South and East Asia, and the Middle East [9,88]. These vegetables not only play an essential role in shaping geographical and cultural diets, but they also provide significant health benefits when incorporated functionally into everyday dishes. Different methods for extracting AITC from such plant materials have been developed, which primarily include mechanical extraction by grinding or pressing grated or macerated mustard seeds or roots, and chemical extraction [65,89].

The naturally produced AITC is found to be bioavailable, although levels accessible biochemically are impacted by a food matrix that may involve the presence of other dietary components; thus, often higher biological effects are typically gained through a higher intake of crude plant materials [90]. It is also reported that additional health benefits, or greater effects achieved from crude mixtures of bioactive compounds, could exceed an equivocal increase in allyl isothiocyanate concentration by using these vegetables or derived materials [1,91]. As a result, allyl isothiocyanate’s health benefits are difficult to compare with other bioengineered forms given their uniqueness [64]. As a further potential variable, the amount of allyl isothiocyanate available may depend on compositional variations related to factors such as geographical location, ecological conditions, agronomical species, growing seasons, storage stability, and cooking impacts, among others [51,92]. It is therefore relevant to underscore the consumption of fresh, raw, or minimally processed Brassicas to increase awareness of natural food sources rich in allyl isothiocyanate for the enhanced health of different populations [47,48].

### 3.2. Synthetic Production

The synthetic production of AITC has family-level relevance for the production of organosulfur compounds and other isothiocyanate compounds [93]. It is noted that AITC is produced using chemical synthesis to facilitate industrial-scale production methods and promptly provide a 100% artificial product for its focused-synthesis applications [87]. Synthetic methods for the production of isothiocyanates are reviewed, which could equally be performed to prepare AITC [94].

This study summarizes the synthetic routes of AITC [95]. Highlighted routes include: the treatment of allyl amine with carbon disulfide followed by dehydration treatment with phosphorous oxychloride; aqueous-phase nucleophilic substitution of an allyl chloride reagent within situ-generated thiosulfate anion, followed by deprotonation and thermal fragmentation; the click synthesis of AITC via the reaction of allyl olefins with tosyl aside in the presence of copper salts; and the direct catalytic dehydrogenation of 3-buten-2-ol isomers in combination with the alkane molecular sieves with molecular H2 gas [94]. Organic chemistry literature highlights the importance of process optimization under the thermodynamically and kinetically controlled regimes of reaction to control this process. Specification-quality AITC is likely to be crude distillation generate-able, especially considering crude specifications include primary purification requirements of being ‘>80% allyl isothiocyanate’ [96]. Future studies in the field are expected to use the Intramolecular Dynamics (IMD) mechanism of the fatty acid synthase (FtSAg2) to enhance the efficiency of the reaction process [97].

In the future, this procedure with modified FtSAg2 complexes as catalysts could be a better way to produce AITC from a tissue inhibitor of metalloproteinases (TiA) without using any solvent [98]. From an industrial point of view, the major limitation in using synthesized AITC for food applications, i.e., pharmaceuticals, antifungal agents, and food additives, is that during some reactions, there is a significant advantage of using natural materials in producing AITC [99]. High-yield and cost-effective synthetic methods for natural AITC sources (NAT) supply should be developed [96]. For example, the AITC yield of the β-elimination of the enzymatic L-ER (the type of enzyme involved in the synthesis process) through targeted natural sources (T-NATs) was 88.4% [97]. In addition, extracted AITC into freeze-dried powder gives a high-quality product with long permit periods and is an eco-friendly extraction method [100].

## 4. Analytical Techniques for Detecting AITC

Several food and biological properties of this phytochemical support the need for accurate identification and quantification [47]. When considering organic decomposition, the volatile compound extracts the maceral proteins, making them easily digestible and bioavailable for use in the body, and is known to lack numerous levels of pharmacological activities that can prevent various types of diseases [76]. Therefore, AITC detection is carried out in different matrices including water, vegetables, as well as processed foods [88]. So far, AITC detection has been primarily conducted for raw foods, while its quantification for microgreen foods made with crops such as flax, radish, broccoli, and mustard sauces is rarely reported [79]. Currently, there is no standard, sensitive, and precise analytical method for detecting and quantifying AITC in natural biological samples [65].

Several analytical techniques are currently available for the detection of AITC, each of which has value based on the inherent characteristics [101]. Gas chromatography-mass spectrometry, high-performance liquid chromatography, and several hybrid methods are often utilized as detection methods because they provide the most accurate and sensitive measurements of compounds from biological samples and natural extracts [61,102]. Although having invaluable analytical power, each method has unique benefits and limitations and should be chosen for the specific target of investigation [103]. Furthermore, sample cleaning methods, such as extraction and purification using a variety of solvents in single or mixtures and several solid-phase materials, enable more accurate determinations [104,105]. The formulation of standard good practices for detecting AITC is critical for reproducibility [106]. A case study on food- and biological-sample analysis using the preferred method is then introduced, and future trends in analytical technology that may improve the detection of AITC in plants and microgreens are discussed [107,108].

## 5. Applications in Food Industry

Freshly produced allyl isothiocyanate was examined for its functional properties, and identifying potential health benefits in humans is well documented [37]. The degradation pathways, such as hydrolysis, oxidation, and polymerization that AITC undergoes can cause instability, so its fresh production is essential to maintain its biological activity and therapeutic effects [109]. It may also find numerous applications within the food industry [110]. For instance, it has been proposed as a natural preservative, with documented effects on the most common microorganisms causing disease in stored food [40]. In addition, AITC can also act as an effective flavoring agent for many kinds of food and food products, while enhancing their sensory qualities [110]. Given this growing consumer demand for food safety and convenience, researchers are studying novel methods to improve the shelf life of processed foods [44]. AITC has been particularly effective against common spoilage organisms like yeast and molds because of its wide spectrum of antimicrobial activity [39]. It is primarily used in processed meats like hams, turkey, and bacon, as well as in cooked meats like marinated or fajita chicken, and to develop unique sauces [111]. The exerted spectrum of activity was cited due to the uniqueness of AITC in damaging yeast cell membranes and inhibiting the growth of molds, as well as slightly reducing the growth of some acid-tolerant spoilage bacteria [112]. It is recognized as follows: “Generally recognized as safe for use in foods following good manufacturing practice.” It has also been assigned as a food additive [44]. To improve the antimicrobial effect, AITC can be used in combination with other natural antimicrobials and prebiotics in food [112]. Unfortunately, the application of AITC in fresh fish may generate a pungent taste or excessive odor, thus decreasing consumer acceptance of the fish products [111]. Although the short-term application of AITC in food can reduce the microbial content, it is only effective for a short period, as microbes may accumulate progressively over time despite the accumulation of AITC in the packaging [110]. The use of AITC as a main active ingredient to study combination preservation technology for increasing the shelf life of food for an extended period is urgent [112] Table 3.

### 5.1. Food Preservation

Nowadays, a wide range of methods are used to lengthen the shelf life of several food products [37]. However, the allies found in mustard produce isothiocyanates, such as AITC, which have considerable promise as natural preservatives [110]. These compounds are generated from their corresponding glucosinolate precursors when the plant tissue is ruptured, giving rise to their unique flavor [44]. Furthermore, AITC has potent antimicrobial activities and can hinder the development of an assortment of foodborne pathogens [39]. In cells, AITC exerts its bactericidal effect by breaking the integrity of the cytoplasmic membrane [35]. In addition, adenosine-triphosphate levels and the integrity of both ribosomal and genomic DNA are significantly reduced due to the disassembly of the ribosomes [84].

Research has shown that the incorporation of AITC in various foods results in an increase in their shelf life [1]. For instance, fish cakes treated with AITC were free from spoilage by a range of foodborne pathogens and lactic acid bacteria for 30 days when stored in modified-atmosphere packaging at 4 °C [113]. Equally, additions of AITC to hamburger meat have been successful in microbial inhibition [114]. For cheddar cheese, it was shown that AITC increased the shelf life of the item by at least 10 weeks at refrigerator temperatures [110]. The use of AITC as a preservative must comply with the current safety regulations [113]. Food producers who utilize synthetic nitrite and nitrate should require artificially ‘cured’ labels for their products [115]. Growing demand, on the part of consumers, for clean-label products makes the application of natural preservatives increasingly attractive for the food industry [84,114].

### 5.2. Flavoring Agent

Allyl isothiocyanate can be used as a flavoring agent in the food industry [1]. This compound possesses a strong pungent aroma with specific sensory characteristics [116]. It is beneficial for creating distinctive and characteristic flavors in various applications [5]. In culinary applications, allyl isothiocyanate contributes to the overall taste of sauces and condiments [116]. Different sauces incorporated with allyl isothiocyanate were sensory evaluated by consumers [102]. Their findings indicated that the greatest preference was given for a pungent, sharp, mustard-like flavor, color, and appearance in product formulations [117]. Other results and studies further confirmed the potential of allyl isothiocyanate as a sensory ingredient suitable for certain novel food products [84]. A major challenge in the application of isothiocyanate was generally achieving the balance right and not overpowering other flavors [110].

As the flavoring is intended to provide a mustard-style flavor, other similar flavored products must also be considered during formulation [48]. Regulations on flavoring substances generally specify permitted upper levels that may be used in various food and beverage applications [3]. Food products are evaluated and approved after application for derogation [1,6]. Even though AITC is a naturally extracted compound, its use is controlled by regulation, including public health and toxicological aspects [12,118]. The test for safety is based on the assessment of the specific components for the general description of chemical substances and specific properties of AITC due to potential hazards under normal conditions of use in food flavorings, as well as those hazards that can occur under foreseeable conditions of misuse [49]. The use of flavor and fragrance in the food industry is also controlled by standards concerning dermal sensitization, with a concentration limit of 0.1% in a final topical formulation [119].

In recent years, there has been an encouraging trend in the industry and also from the consumer side to use natural agents as flavorings [120]. It is widely claimed that consumers generally perceive natural foods to be more cognitively permeable as they carry a lesser degree of artificiality and hazards not intrinsic to natural products [121]. In addition, the advantages of the nutrition and healthfulness qualities of natural products over synthetic ingredients are some of the main reasons many food companies have shifted from using synthetic flavorings, owing to consumer concerns about food safety and health [122]. The wide array of flavorings used in the formulation of oral medication for both humans and animals aids in limiting the bad taste and smell of certain drugs [123]. The use of kasundi and mustard sauce containing AITC on the market demonstrates their potential use, based on their long shelf life and stability [120]. The market, especially in food preservation treatments, has given the product excellent appeal to consumers [124]. Companies can produce better economic innovativeness in a cooperative venture, creating added value for consumers and businesses alike [125]. While AITC presents itself as a promising natural alternative to some synthetic food additives, economic realities pose challenges. Extracting AITC from natural sources often incurs higher costs compared to synthesizing artificial additives. Furthermore, AITC’s volatile nature necessitates careful handling and storage, adding to the overall expense. The current market for AITC as a food additive may also be limited, potentially impacting on its economic viability for manufacturers. Finally, ensuring compliance with food safety regulations for AITC use can involve additional costs for testing and documentation [126].

## 6. Potential Applications in Medicine

Currently, AITC is considered the main ingredient responsible for the therapeutic benefits of mustard oil, which is commonly used in Ayurvedic and traditional Chinese drugs [49]. This compound is also used topically and included in the composition of many pharmaceutical formulations. It seems that AITC could become an active ingredient in modern therapy, and its potential could be much more extensive than now understood, especially since its active form can be obtained from endogenous glucosinolates delivered under certain digestive conditions [18]. Pharmacological studies indicate that this compound has anti-inflammatory, analgesic and antimicrobial effects [127]. AITC, like other ITCs, has been shown to display anticancer activity in many basic in vitro and in vivo studies [128]. A potential therapeutic benefit lies in the possibility of further exploring the apoptosis-inducing mechanism and the influence of AITC on carcinogenesis and other biological processes [129]. It offers new opportunities for the enhancement of cancer treatment strategies, including both pharmacological therapy and integrative medicine [130]. In particular, new, innovative potential medical applications of AITC can be expected in the context of interdisciplinary and translational sciences [131]. It should be noted that the use of AITC in medical products is associated with many regulatory issues, and the need to execute clinical trials to prove its safety and to confirm or refute its expected effect is mandatory [132]. Pharmacokinetic studies are also crucial, however, they have not been carried out so far [127].

### 6.1. Pharmaceutical Formulations

AITC, a naturally occurring, odorous, volatile, and colorless liquid, is a perfect candidate for use as an active substance in pharmaceutical formulations [47]. This saturated aliphatic isothiocyanate, the main component of mustard oil, is recognized to have a part in chemo-reduction and exhibits antibacterial, antifungal, and anti-inflammatory properties [3]. Good pharmacodynamic (low acute oral toxicity, high sensory threshold to red mustard oil, and acceptance for potential users) and pharmacokinetic properties (absorption mainly occurring in the oral cavity and the gastrointestinal tract’s top portion or in the site of action, and an intensive process of metabolic transformations) and pharmacotherapeutic potential to support the therapy of chronic diseases indicate the potential to exploit the opportunities presented by freshly synthesized AITC in the treatment of all types of chronic diseases [70]. Thus, AITC is suitable for the development of innovative dosage forms intended for oral administration to act at a target location [18]. The therapeutic applications of AITC are limited by the stability of its molecules, their low water solubility, and several quality parameters that must be addressed for a given drug to be introduced onto the market [67]. In pharmaceutical production, the main focus is on the development of immediate-release dosage formulations involving tablets or capsules [129]. Some of the AITC dosage forms used at present include a product recommended for use in the form of a nutritional supplement in the treatment of Helicobacter pylori infection, as well as mustard plasters and mustard baths [133]. In general, laws establishing protocols for the approval and oversight of pharmaceuticals intended for human and veterinary use apply to the development of novel medications [134].

In summary, the review of the presented pharmaceutical, preclinical, and clinical parameters indicates that newly synthesized AITC is suitable for use as an active substance provided that its stability in solid- or liquid-media in vitro, in addition to in ex vivo and in vivo studies, is determined [135]. Based on the conducted study, it is postulated that the data indicates AITC from mustard oil is a suitable active pharmaceutical ingredient for oral delivery to the lower gastrointestinal tract for treatment and future clinical studies to evaluate its therapeutic benefit as a potential natural chemo-preventive supplement [67]. The use of AITC powder suited to different severity liquids should be considered, especially the possibility of implementing immediate release tablets. An additional reason is that the potential of this substance to enhance the effectiveness of the standard therapy for type 2 diabetes could be used, for example, in the modified release formulations that are under development. The remaining numbers were provided for comparison purposes based on the obtained data; these represent different substances and do not characterize the drugs being studied [133]. The dose of AITC that seems to be effective as a therapeutic/adjuvant substance is less than 0.6 mg of this compound in the dose, valid as a medicine or for food/nutritional supplements, and accepted for use as safe [133]. It is postulated that further studies should be carried out by pharmaceutical/clinical institutions to determine the maximum dose of AITC that is safe and at the same time has chemo-preventive effectiveness as an oral therapy among patients suffering from different forms of metabolic syndrome [10].

### 6.2. Therapeutic Potential

Given the potential therapeutic applications of AITC, its role as a natural product in preventive and combative therapy for some chronic diseases, especially those with inflammation in etiology or strong associations with inflammation, such as cancer, should deserve prompt interest [48]. Indeed, an increasing number of studies are discovering the anti-proliferative, anti-inflammatory, and pro-apoptotic actions of AITC in tumor cells of different origins [136]. Consequently, the underlying mechanisms are being elucidated, focusing to a great extent on the modulation of cellular signaling pathways—mainly kinases and transcription factors—in addition to the genetic transcription machinery [9]. Furthermore, evidence of mitigation or blockade of these pathologies by AITC and AITC-rich products is progressively reported, either in vitro, ex vivo, or in clinical settings working on various tumor types [137]. The findings of these studies offer new prospects for designing more effective medical therapies [138]. Although not yet definitive, these competing works highlight that the use of AITC will require a careful definition of doses and administration regimens [139]. The therapeutic success of these agents, though still far from reach, will rely on the optimization of such parameters to improve compliance concerning harmful secondary effects of AITC, although those derived from an exaggerated reaction to its intake also drive the attention of ongoing toxicological research [140,141]. In addition, Cruciferous vegetables contain high concentrations of thioglucosides that are metabolized to thiocyanates. These compounds inhibit iodine transport and the incorporation of iodide into thyroglobulin, therefore increasing TSH secretion and thyroid cells proliferation [142].

## 7. Toxicological Considerations

Safety is an important issue for any compound destined for food, feed, or pharmaceutical applications [143]. The potential toxicological effects of AITC should be a primary consideration when determining its application and optimal dosage [144]. To date, only a few adverse effects have been observed upon the use of AITC [63]. Generally, huge doses of AITC can result in potential health risks, for example, damage to the oral mucous membrane, pulmonary edema, or lesions to the liver, kidneys, and reproductive organs [93]. Various research conducted in vitro, in vivo, and in different model systems demonstrates the potentially toxic effects of AITC following acute and chronic exposures [9]. Information on the toxicological effects of AITC is well documented, and it is generally known what chemical mechanisms underlie its biological effects [51,144].

It has been documented that AITC is toxic to biological systems [83]. In primary skin cells, AITC induced necrosis and apoptosis at lower doses and only cytotoxicity at higher doses over 2 h [145]. In the liver of rats, AITC produced specific adverse effects in response to different exposure times and doses [146]. In pregnant rats and developing embryos, AITC has been found to induce reproductive and developmental toxicity, especially via oral administration [143]. Any decision on the use and application of AITC in the food and feed sectors should be based on safety evaluation tests [28]. These tests need to comply with good laboratory practices and safety assessment protocols for use in pharmaceuticals and foodstuffs [147]. The allowable concentration of AITC that can be safely utilized is strictly evaluated and regulated. However, the mechanisms of toxicity can vary between animal species based on differing digestive systems and steroid metabolism in rodents [148]. Furthermore, human beings can react depending on variation between individuals [149].

### 7.1. Safety Assessment

The allyl isothiocyanate (AITC) safety has been assessed by different regulatory agencies responsible for ensuring consumers are protected from unsafe or adulterated food. General specifications, procedures, and guidelines governing the use of allyl isothiocyanate in food and supplement products are provided [83]. A version of GRAS (Generally Recognized as Safe) status has been designated for allyl isothiocyanate when formulated in polymers for use in pharmaceutical products [115].

Scientific data on the toxicology of exposure to excessive levels of AITC have been reviewed by relevant health authorities [6]. A review of allyl isothiocyanate was previously reported using results from acute, sub-chronic, chronic, reproductive, and developmental exposure data in preclinical animals and human subjects [144]. A risk assessment model for allyl isothiocyanate effects on the axonal reflex in rats has been proposed [150]. The toxicology of allyl isothiocyanate used in the manufacture and formulation of polymers for the time-controlled release of AITC in food is not completely known [151]. According to present in vitro and in vivo results, concentrations 10,000-fold greater than the anticipated total dietary exposure are possibly acceptable in animal testing of formulated polymers containing AITC [152]. Currently, treat-and-release studies are planned with AITC food-contact polymers. Allergenic responses to it are relatively rare, and little is known about the acquired immune response in the general population consuming AITC [139].

Further, despite a general agreement that AITC administration produces an aversive response and associated cardiopulmonary changes in humans, currently, there is relatively limited data from controlled long-term exposure human studies to truly understand the consequences of the AITC phenomenon [153]. In this regard, some clinical research may be warranted in the future, particularly in identifying populations most likely to develop sensitivity, more accurate assessment of exposure data during ordinary food consumption, and definitive studies of the long-term effects of AITC neurogenic exposure [154]. Evaluated outcomes should include the culturally relevant food intake patterns and perceived changes concerning their exposure thresholds, meal frequency, and tolerance to chronic irritation [155]. Preclinical models, such as chronic dietary exposure studies in dogs, will be part of future risk assessment studies of this compound [156]. The clinical development objective is to determine bases of safety in human volunteers, who briefly ingest small quantities of AITC incorporated into various food products [157].

### 7.2. Adverse Effects

Several reports have raised attention to the adverse effects of AITC contamination in foodstuffs. A study documents gastrointestinal, respiratory, and skin reactions, as well as liver dysfunction in four subjects one hour after consuming pure mustard oil containing 1.2% AITC [1,5]. Under in vivo conditions, AITC might form a very soft acid, inactivated in the upper airways and gastrointestinal system to some extent, as well as being produced in situ by plant endogenous processes [158]. The respiratory system and skin are easy targets for exposure because they lack protective filters [39]. Ingestion, particularly when AITC is dissolved in beverages, is trivial concerning vapor aspiration because there is a qualitative correspondence of solubility in beverages and water [159]. The adverse effects reported in subjects are consistent with the intensity of transient symptoms of mild to acute exposure [160].

There are several potential explanations for the negative consequences observed in people [1]. Hypersensitivity is not easily deduced because there is a lack of evidence of an allergic reaction mediated via IgE antibodies to date, even in mustard seed workers with bronchial asthma [161]. AITC has marked irritant properties, but individual variability must be taken into account, so some subjects appear to be more sensitive than others or have a stronger asthmatic response to AITC [154]. Thus, the risk associated with exposure to allyl isothiocyanate needs to be carefully considered [161]. However, expert assessments have reported long-term adverse effects of mustard condiment oil, and the reports associated with horseradish allyl isothiocyanate contained much noise, whereas a relative calmness was observed regarding patents concerned with allyl isothiocyanate, its preparations, and uses [162]. As a consequence, no changes in regulations were recorded until 2004 [161]. Transparency is essential when using allyl isothiocyanate, which is known to be potentially harmful [163]. Risk communication should be provided to users within the framework of very low general migration limits at 0.02 mg allyl isothiocyanate per kilogram of food, a possible temporary safety measure. An example of a food matrix with an AITC concentration limit is mustard-flavored potato chips, where the maximum AITC concentration allowed is 0.1% [164]. It is assumed that allyl isothiocyanate behaves as an irritant sensitizer when use conditions significantly reduce vapor pressure, in addition to conditions creating hand-skin damage, handling low boiling point products, and other physicochemical properties of allyl isothiocyanate [165]. It is advised that when handling AITC, one should wear the proper personal protective equipment (PPE), such as gloves, overalls, shoes, goggles, or face protection, and adhere to the rules of good occupational hygiene [166].

## 8. Regulatory Status and Guidelines

In the U.S., the concentration of allyl isothiocyanate must not exceed 0.1% [167]. AITC is categorized as a “natural flavoring” which is generally recognized as safe when used within the regulation level established [86]. In the European Union, the maximum amount of allyl isothiocyanate that can be used in food and medicinal goods is 5 mg/kg [85]. The commercial formulation of allyl isothiocyanate could be inconsistent; the standardization of commercial formulation from extraction is required to meet the safety of consumers [168]. Several regulatory requirements must be considered during the development of commercial formulations [169]. Health, safety, security, environment, and public relations guidelines exist as a framework for the safe use of AITC [13]. Responsible parties in the industry are obligated to comply with these guidelines, thus ensuring consumer welfare and addressing pressure from industry stakeholders [61]. The mismanagement of natural compounds resulting from a growing conscientious interest in active disclosure has now also afforded a critical opinion [170]. If one were to look to the horizon, companies have initiated aspects of R&D to the direct effect of what may be reasonable or acceptable for end-use AITC [28].

## 9. Future Research Directions

Despite the roadblocks when exploring laid-out horizons for AITC, in vivo, in vitro, and cell assays have been thoroughly studied [21]. Recently, many in vitro and cell assays focusing on signaling transduction, genetics, and proteomics have provided novel ideas for AITC research [94]. However, the long-term health outcomes are still not clear, especially regarding AITC bioavailability [81]. Therefore, we suggest that a more comprehensive investigation be conducted to better understand the potential health applications of AITC, such as pharmacology, toxicology, nutrition, and more [94]. The last roadblock depicts the state of information possessed by researchers on AITC, a relatively powerful antioxidant recognized as a radical scavenger [93]. It is generally accepted that the searches on the protective features of AITC have just begun to scratch beneath the superficial alternatives that maintain its diverse actions [3]. The explanation and data gathered from recent literature, along with the distinct descriptions of metabolism and other characteristics, have unleashed growing interest in newly developed computation-based systems that predict molecular interactions and bioactivities that still demand verification [171]. Therefore, significant insight into interdisciplinary research in comparative nutrition, pharmacology, and toxicology is important to solve a multitude of questions about the biological features of AITC and alternate therapies prompting in vitro, in vivo, and cell-regulated clinical tests standardized to authenticate usage in human and animal settings [20]. These approaches and complementary upcoming options can accelerate the study and enhance therapeutic concepts to be maintained in clinical findings [172]. Therefore, future opportunities include findings that greatly improve our comprehension of intuitive mechanisms related to therapeutic findings that distinguish AITC’s potential studies at multiple stages [62].

## 10. Conclusions

AITC has sparked widespread interest due to its various biological actions, which include strong antioxidant, anti-inflammatory, antibacterial, anticancer, and cardiovascular capabilities. This compound offers promising potential in several fields, particularly in food preservation, medicine, and enhancing food quality through natural means. Furthermore, the potential to extend the shelf life of perishable foods makes AITC an important tool for food production, meeting rising customer demand for natural additives.

However, AITCs still face several challenges related to AITC’s volatility, dosage optimization, and regulatory considerations. We strongly recommend that future research be held to enhance the stability and efficacy of AITC-based formulations. In conclusion, in a world increasingly leaning toward discovering alternative sources, exploring AITC is considered a great opportunity as a natural alternative source. Our findings underscore AITC’s role as a versatile bioactive compound with known potential to support human health and the sustainable food industry.

## Figures and Tables

**Table 1:** Health-promoting properties and mechanisms of action of allyl isothiocyanate.

Health Benefit/Property	Mode of Action	Research Method	Reference
Antioxidant Properties	Freeze-drying preserves properties; hot-air drying enhances activity	Not specified	[16]
Antimicrobial Properties	Broad-spectrum activity against bacteria and fungi; disrupts membrane potential and enzymatic function	In vitro, animal studies	[17,18,19,20,21,22,23,24,25,26,27,28,29,30,31,32,33,34,35,36,37,38,39,40,41,42,43,44,45,46]
Anti-inflammatory Effects	Suppresses cytokines (e.g., TNF-α, IL-6); binds to NF-kB, COX-2, and iNOS; reduces pro-inflammatory mediators	In vitro, animal studies	[47,48,49,50,51,52,53,54]
Anticancer Potential	Induces apoptosis; cell cycle arrest at G2/M phase; downregulates Bcl-2; upregulates Bax; modulates signaling pathways	In vitro, limited population studies	[47,48,49,50,51,52,53,54,55,56,57,58,59,60,61,62,63]
Cardiovascular Benefits	Enhances endothelial function, reduces cholesterol, and improves lipid profiles; antioxidative and anti-inflammatory effects	Animal studies, human pilot studies	[1,2,8,64,65,66,67,68,69,70,71,72]
Preservative Applications	Inhibits growth of foodborne pathogens and spoilage agents; synergistic effects with other antimicrobials	In vitro, food matrices	[34,35,36,37,38,39,40,41,42,43,44,45,46]
Immune Modulation	Immunosuppressive effects; influences cytokines, macrophages, and dendritic cells	Animal studies	[47,48,49,50,51,52,53,54]

**Table 2:** Antibacterial activity of AITC and the mechanism of action.

AITC Source	Reasonable Bacteria	Mechanism of Action	Reference
*Wasabia japonica*	*S.aureus* *E. coli O157:H7*	*E. coli* O157:H7 cell membrane integrity is impacted by AITC.	[73]
Synthetic	*C. jejuni* *E. coli O157:H7*	AITC inhibits the activity of *E. coli* O157:H7 thioredoxin reductase and acetate kinase by reacting with the enzymes’ sulfhydryl groups. AITC prevents the synthesis of Shiga toxin by EHEC *E. coli*	[74]
Synthetic	*E. coli CECT 434* *P. aeruginosa* ATCC 10145 *S. aureus* CECT 976	The cell’s membrane integrity of *S. aureus* CECT 976, *P. aeruginosa* ATCC 10145, and *E. coli* CECT 434 is impacted by AITC.	[37]
Synthetic	*MRSA*	In comparison to the other ITCs, the aliphatic structure of AITC inhibits MRSA growth.	[75]

The table illustrates the antimicrobial activity of AITC against bacterial strains such as *Staphylococcus aureus* (*S. aureus*), *Escherichia coli* (*E. coli*), *Campylobacter jejuni* (*C. jejuni*), *Pseudomonas aeruginosa* (*P. aeruginosa*), and Methicillin-Resistant Staphylococcus aureus (MRSA).

**Table 3:** Applications of AITC in the food industry.

Industry	Benefits	Origin of AITC Used	Short Description of Application	Reference
**Food Industry**	Natural preservative, flavoring agent, and antimicrobial activity.	Extracted from mustard seeds or synthesized.	Extends shelf life of processed foods, prevents spoilage by microorganisms, and adds distinctive flavor to sauces, condiments, and other food products.	[37,40,110]
**Food Preservation**	Increases shelf life of perishable items like fish, meat, and dairy products.	Freshly produced AITC.	Effective against spoilage microorganisms, used in modified atmosphere packaging, and combined with other antimicrobials to enhance preservation.	[113,114,115]
**Flavoring**	Provides distinctive mustard-like flavor and enhances sensory qualities of food.	Derived naturally or synthetically.	Used in sauces, condiments, and beverages; sensory evaluations reveal consumer preference for its pungent and sharp characteristics.	[116,117,118]

## Data Availability

No new data were created or analyzed in this study. Data sharing is not applicable to this article.
